# Assessing retinal hemorrhages with non-invasive post-mortem fundus photographs in sudden unexpected death in infancy

**DOI:** 10.1007/s00414-023-02964-9

**Published:** 2023-02-23

**Authors:** Jean-Baptiste Ducloyer, Cloé Scherpereel, Thomas Goronflot, Guylène Le Meur, Pierre Lebranchu, Frédérique Jossic, Virginie Scolan, Mathilde Ducloyer

**Affiliations:** 1 Nantes Université, CHU Nantes, service d’ophtalmologie, Nantes, France; 2 CHU Grenoble, service de médecine légale, Grenoble, France; 3 Nantes Université, CHU Nantes, Pôle Hospitalo-Universitaire 11: Santé Publique, Clinique des données, INSERM, CIC 1413, F-44000 Nantes, France; 4 Institut d’histopathologie (IHP Group), Angers, France; 5 Nantes Université, CHU Nantes, service de médecine légale, Nantes, France; 6 Nantes Université, CHU Nantes, INSERM, CIC 1413, 44000 Nantes, France

**Keywords:** Sudden unexpected death in infancy, SUDI, Post-mortem, Retinal hemorrhages, Abusive head trauma, RetCam

## Abstract

**Introduction:**

In the case of sudden unexpected death in infancy (SUDI), eye examination is systematic to detect retinal hemorrhages (RH) that are a crucial hallmark for abusive head trauma (AHT). The aim of this study is to assess the ability of non-invasive post-mortem fundus photographs (PMFP) to detect RH in case of SUDI.

**Methods:**

Bicentric retrospective analysis of consecutive cases of SUDI under 2 years of age were managed by two French SUDI referral centers with PMFP by RetCam (Clarity Medical Systems USA). PMFP were reviewed randomly, twice, by three independent ophthalmologists blinded for clinical data.

**Results:**

Thirty cases (60 eyes) were included. Median age was 3.5 months (interquartile [1.6; 6.0]). No child died of AHT. Image quality was sufficient to assert presence or absence of RH in 50 eyes (83%). Sufficient quality rate was significantly higher when the post-mortem interval was inferior to 18 h (91%, 42/46) as opposed to over 18 h (57%, 8/14, *p*=0.0096). RH were found in six eyes (10%), four children (13%), with excellent inter and intra-raters’ concordance (Cohen’s Kappa from 0.81 [0.56–1.00] to 1.00 [1.00–1.00]).

**Conclusion:**

PMFP can detect RH in case of SUDI and is a relevant systematic screening test to be carried out as soon as the deceased child arrives in the hospital. It can decrease the need of eye removal for pathological examination, but further studies are needed to define the best decision algorithm.

**Supplementary Information:**

The online version contains supplementary material available at 10.1007/s00414-023-02964-9.

## Introduction

Each year in Europe, 35 infants per 100,000 live births die suddenly and unexpectedly before the age of one: sudden death in infancy (SUDI) is the first cause of death after the neonatal period in France [1]. Sudden infant death syndrome (SIDS) is defined as the sudden death of an infant under one year of age, which remains unexplained after a thorough case investigation, including performance of a complete autopsy, examination of the death scene, and review of the clinical history [2, 3]. When pediatricians and forensic pathologists are confronted to SUDI, they must thoroughly research a cause of death before concluding SIDS. Abusive head trauma (AHT) is defined as an injury to the skull or intracranial contents of an infant under 5 years of age, due to inflicted blunt force impact and/or shaking [4, 5]. Infanticide by fatal AHT is a special cause of SUDI firstly because it is difficult to suspect and to confirm, and secondly because its recognition will lead to a judicial enquiry [6]. As retinal hemorrhages (RH) are a crucial hallmark for AHT (sensitivity=75% and specificity=94%) [7], the thorough case investigation must include systematic eye examination.

To date, there is no consensus on the best approach to detect RH. The American Academy of Pediatrics recommends post-mortem eye removal in case of SUDI under 5 years of age that have not clearly died of witnessed severe accidental head trauma or readily diagnosed systemic medical conditions [8]. However, the French Haute Autorité de la Santé (HAS) recommends systematic post-mortem fundus examination [9]. The relevance and the protocol of post-mortem eye removal as part of the autopsy have been well described [10]. Conversely, reports of post-mortem fundus examination are very rare and no protocol has been yet validated between endoscopy [11, 12] or indirect ophthalmoscopy [13]. Wide field fundus camera such as RetCam (Clarity Medical Systems USA) is the gold standard for the acquisition of retinal images in suspected cases of AHT in living children [14], but this method has never been described in deceased children. Assessing the capacity of post-mortem fundus photographs to detect RH is a major issue because it is non-invasive, it does not need eye removal, it allows screening of a wider range of children without problems of acceptability and availability, and the response is immediate.

The aim of our study was to assess the capacity of post-mortem fundus photographs (PMFP) by Retcam to detect RH. We hypothesized that RetCam PMFP can detect RH and may become a valuable screening test complementary to pathological examination.

## Methods

This bicentric retrospective study was conducted in two French University Hospitals. Inclusion criteria were: SUDI under 2 years of age, PMFP realized by RetCam and available for reinterpretation. The definition of the cases of SUDI followed the international definition, namely the death of an apparently healthy child under the age of 1 year, with an extension to the children up to 2 years of age, as recommended by the French recommendations for the management of SUDI [2, 3, 15]. The following clinical data were collected from medical records and from the French SUDI registry (Observatoire National des Morts Inattendues du Nourrisson registry; OMIN): age at death, sex, post-mortem interval between death and PMFP, final diagnosis after complete case investigation. Post-mortem interval (PMI) used in our analysis was the mean between minimum PMI (interval between the finding of the deceased child and the PMFP) and maximum PMI (interval between the last observation of the living child and the PMFP). Complete post-mortem investigations included: a complete external examination of the skin and biometrical measurements, biological samples (virological and bacteriological analyses of blood, cerebrospinal fluid, urines, feces; complete blood count; biochemical markers on blood and cerebrospinal fluid), genetic samples (with the agreement of both parents), whole body post-mortem imaging (X-ray on the skeleton and post-mortem computed tomography and/or post-mortem MRI), fundus, toxicological analysis, chromatography of organic acids in urine, forensic or scientific autopsy with pathological examination of the organs, solicited by the prosecutor or after acceptance by the parents. A detailed interview of the parents was systematically realized by the medical staff and/or the police officer, to collect all necessary data on the medical background of the family and the child and the context of the death. The prosecutor was warned to check the criminal records of the parents and/or family. The determination of the cause of death was established by a multidisciplinary staff which summarized the opinion of the forensic pathologist, the pediatrician of the referral center, the nurse of the referral center, the ophthalmologist and the radiologist.

RetCam (Clarity Medical Systems, USA) is a digital wide field camera developed to assess pediatric eye diseases. It allows a dynamic examination on a large screen and the acquisition of videos and photographs. The examination lasted 5–10 min. The eyelids were kept open with an eyelid speculum. We did not use dilating eye drops as the pupils cannot react after death and are already slightly dilated. An ophthalmic gel was instilled on the eye and added if needed during the examination to maintain the optical contact between the cornea and the camera. If needed, the superficial corneal surface was gently rubbed with a microsponge to remove edematous and cloudy corneal epithelium. For each eye, numerous photographs were acquired to evaluate the posterior pole (macula and optic nerve) and the peripheral retina. The examination was performed either in a hospital room, an autopsy room or in the emergency department, as soon as the child was arrived at the hospital. All PMFP in the center 2 and many PMFP in the center 1 have been performed by forensic pathologists, after a short training period.

For each eye, the PMFP series were collected in an anonymous PDF file. The PMFP series were randomly and independently reviewed by three senior ophthalmologists blinded for all clinical data. For each eye, the following data were assessed: image quality to assert presence of RH, presence of a macular retinal fold, horizontal and vertical dimension of the macular fold (in optic disc diameters), presence of a peripheral retinal fold, and papillary vessels enlargement. To assess the image quality, the examiners answered “yes” or “no” to the question “Was the retina sufficiently visible on the images to determine the presence or absence of RH?”. To help them, they were given a large panel of fundus photographs showing retinal hemorrhages, extracted from reference articles in the literature [8, 16, 17]. If RH were visible, they were classified according to the “traumatic hemorrhagic retinopathy (THR) grading system” [17]. The term “papillary vessels enlargement” referred to the presence of blood in the pre papillary vessels which appear enlarged compared to the other retinal vessels. A second review was completed by the same ophthalmologists at least 1 month later, with different anonymization numbers, in a different random order.

Patients’ characteristics were presented as the median and interquartile range for continuous variables and as effective and percentage for categorical variables. For univariable comparisons, we used Kruskal–Wallis one-way analysis of variance for the former and Fisher exact test for the latter. Missing data were systematically presented. For descriptive purposes, we have chosen to retain the majority decision to summarize evaluators’ assessments. Each PMFP was evaluated 6 times (3 evaluators, 2 series). The quality of the fundus pictures to assess the presence of RH was regarded as sufficient if it was positively evaluated more than 3 times by the assessors, insufficient if it was positively evaluated less than 3 times or discordant if it was positively evaluated 3 times. There was a complete agreement if it was positively evaluated 0 or 6 times. Concordance was measured using Cohen’s Kappa for intra-rater reliability and 2 by 2 inter-raters’ reliability with 95% confidence intervals estimates. Analysis was performed using R version 4.0.4 [18].

The study followed the tenets of the Declaration of Helsinki. Following the French rules on medical research, no institutional review board approval was required because of our study’s non-interventional and retrospective design, and anonymization of the cases. The parents or people who have parental authority gave informed and written consent before inclusion in the OMIN registry.

## Results

**Fig. 1:**
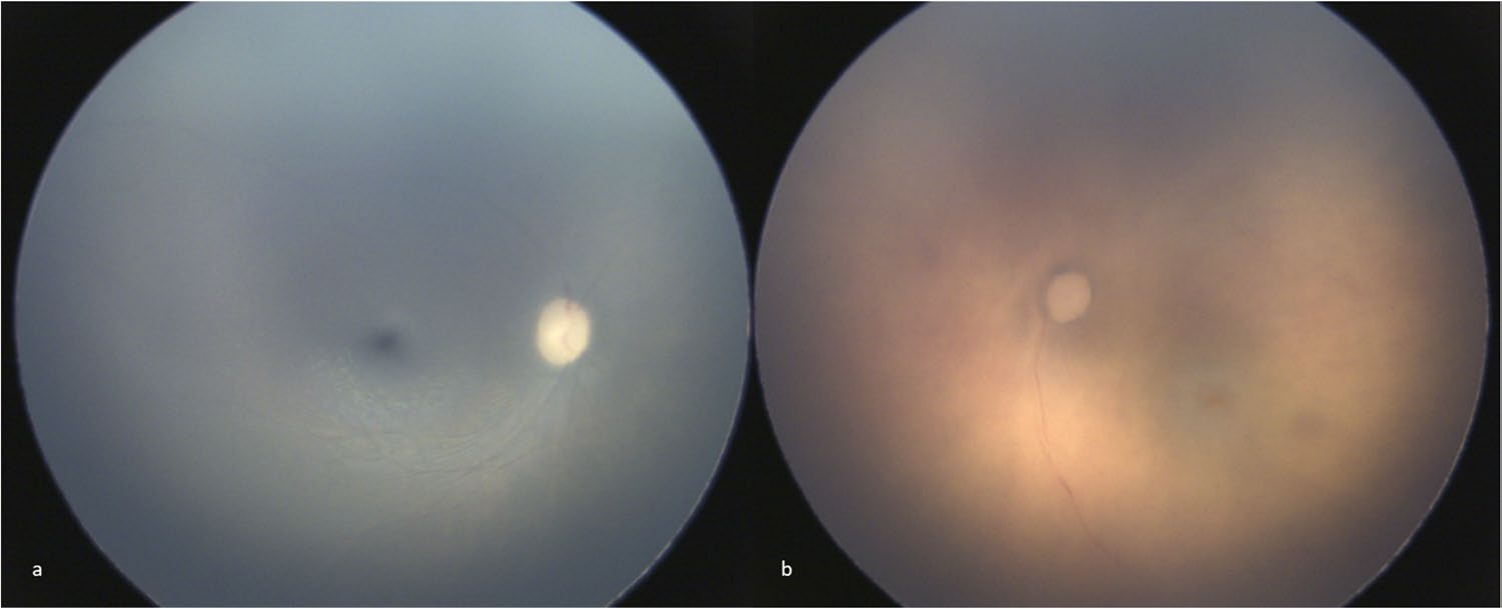
Post-mortem fundus photographs with quality sufficient to assert presence of absence of retinal hemorrhages suggestive of AHT. **a** Right eye of a three-month-old-girl, 4.5 h of post-mortem interval (PMI), undetermined cause of death; **b** Left eye of a 2-month-old boy, 9 h of PMI, cardiac cause of death

Sixty eyes from 30 cases of SUDI between March 2017 and July 2021 were included, 17 girls and 13 boys (Table 1). PMFP was performed either by resident or senior, ophthalmologists, or forensic pathologists. Median age was 3.5 months (interquartile (IQR) [1.6; 6.0]). Regarding the causes of death, the main causes which were retained after all post-mortem investigation was asphyxiation (*n*=13) and SIDS (*n*=5). The cause of death was considered as undetermined in eight cases due to the lack of some post-mortem results at the time of the study. No child died from AHT in our series. Of 60 eyes, image quality was sufficient to assert presence or absence of RH suggestive of AHT in 50 cases (83%) (Fig. 1) and insufficient in 6 cases (10%). The assessment of image quality was completely identical between the six examinations for 45 eyes (75%) but was conflicting in 15 eyes (25%) (Table 2). Of these 15 eyes, 11 were classified as “sufficient” or “insufficient” by the majority and 4 were classified as “discordant” in the absence of a majority. Intra- and inter-raters’ Cohen’s Kappa led to a moderate to excellent concordance when assessing image quality for RH observation (*κ* = 0.41 [0.12–0.70] to *κ* = 0.84 [0.66–1.00], Table 5 – supplemental data). The classification was identical between the two eyes for 27 children (90%). The three groups were similar with respect to age, center, cause of death, year of death (Table 1). Median PMI was significantly lower in “sufficient quality” cases (10.2h [6.3, 13.8]) than in “insufficient quality” cases (19.0h [12.0, 27.6], *p* = 0.04985). Additionally, sufficient quality rate was significantly higher when PMI was inferior to 18 h (91%, 42/46) than when PMI was superior to 18 h (57%, 8/14, *p*=0.0096) (Table 3). This difference was similar in all centers.

**Table 1:** Characteristics of the study population. *SIDS* sudden infant death syndrome

Characteristics		Image quality to assert presence of absence of retinal hemorrhages			
		Total	Insufficient quality	Discord	Sufficient quality
*N*, eyes		60 eyes of 30 children	6 eyes (10.0%) of 4 children	4 eyes (6.7) of 3 children	50 eyes (83.3) of 26 childre**n**
Center, eyes (%)	*Grenoble*	14 eyes (23.3)	1	3	10
	*Nantes*	46 eyes (76.7)	5	1	40
Age in months (median [IQR])		3.50 [1.62, 6.00]	3.50 [2.00, 5.75]	14.50 [4.75, 24.00]	3.00 [1.50, 6.00]
Sex, children (%) (eyes)	*Male*	13 (43.3)	0 (0.0)	1 (33.3) (1)	13 (50.0) (25)
	*Female*	17 (56.7)	3 (100.0) (6)	2 (66.6) (3)	13 (50.0) (25)
Cause of death, children (%) (eyes)	*Asphyxia*	13 (43.3)	3 (75.0) (5)	2 (66.6) (2)	9 (37.5) (19)
	*Undetermined*	8 (26.7)	1 (25.0) (1)	0 (0.0)	7 (29.2) (15)
	*Sids*	5 (16.7)	0 (0.0)	0 (0.0)	5 (20.8) (10)
	*Cardiac failure*	1 (3.3)	0 (0.0)	0 (0.0)	1 (4.2) (2)
	*Infection*	1 (3.3)	0 (0.0)	0 (0.0)	1 (4.2) (2)
	*Lung failure*	1 (3.3)	0 (0.0)	0 (0.0)	1 (4.2) (2)
	*Intoxication*	1 (3.3)	0 (0.0)	1 (33.3) (2)	0 (0.0)
Year of death, children (%) (eyes)	*2017*	*2*	0 (0.0)	1 (33.3) (1)	2 (7.6) (3)
	*2018*	*5*	1 (25.0) (2)	0 (0.0) (0)	4 (15.4) (8)
	*2019*	*7*	1 (25.0) (1)	1 (33.3) (1)	6 (23.1) (12)
	*2020*	*8*	1 (25.0) (2)	1 (33.3) (2)	6 (23.1) (12)
	*2021*	*8*	1 (25.0) (1)	0 (0.0) (0)	8 (30.1) (15)
Post mortem interval in hours (median [IQR])		10.39 [6.49, 17.46]	19.00 [12.02, 27.62]	14.84 [7.08, 22.30]	10.28 [6.26, 13.80]

**Table 2:** Images reviews and agreement rates

Results	Level	Images (*n* = 60)
Image quality to assert presence or absence of retinal hemorrhages, eyes (%)	*Insufficient*	6 (10.0)
	*Discord*	4 (6.7)
	*Sufficient*	50 (83.3)
Complete agreement between the 6 examinations about the image quality to assess retinal hemorrhages (%)	*No*	15 (25.0)
	*Yes*	45 (75.0)
Presence of retinal hemorrhages (%)	*No or quality insufficient*	54 (90.0)
	*Yes*	6 (10.0)
Complete agreement between the 6 examinations about the presence of retinal hemorrhages (%)	*No*	2 (3.3)
	*Yes*	58 (96.7)
Image quality to assert presence or absence of papillary vessels enlargement (%)	*Insufficient*	6 (10.0)
	*Discord*	1 (1.7)
	*Sufficient*	53 (88.3)
Complete agreement between the 6 examinations about the quality image to assess papillary vessels enlargement (%)	*No*	9 (15.0)
	*Yes*	51 (85.0)
Presence of papillary vessels enlargement (%)	*No or quality insufficient*	46 (76.7)
	*Discord*	1 (1.7)
	*Yes*	13 (21.7)
Complete agreement between the 6 examinations about the presence of papillary vessels enlargement (%)	*No*	11 (18.3)
	*Yes*	49 (81.7)
Image quality to assert presence or absence of macular retinal folds (%)	*Insufficient*	4 (7.0)
	*Discord*	4 (7.0)
	*Sufficient*	52 (87.0)
Complete agreement between the 6 examinations about the quality image to assert presence or absence of macular retinal folds (%)	*No*	15 (25.0)
	*Yes*	45 (75.0)
Presence of macular retinal folds (%)	*No or quality insufficient*	18 (30.0)
	*Discord*	4 (7.0)
	*Yes*	38 (63.0)
Complete agreement between the 6 examinations about the presence of macular retinal folds (%)	*No*	16 (27.0)
	*Yes*	44 (73.0)
Horizontal length of macular retinal folds, measured in optic disc diameters (median [IQR])		3.00 [0.38, 4.00]
Vertical height of macular retinal folds, measured in optic disc diameters (median [IQR])		0.75 [0.08, 1.09]
Image quality to assert presence or absence of peripheral retinal folds (%)	*Insufficient*	9 (15.0)
	*Discord*	6 (10.0)
	*Sufficient*	45 (75.0)
Complete agreement between the 6 examinations about the quality image to assert presence or absence of peripheral retinal folds (%)	*No*	18 (30.0)
	*Yes*	42 (70.0)
Presence of peripheral retinal folds (%)	*No or quality insufficient*	45 (75.0)
	*Discord*	1 (1.7)
	*Yes*	14 (23.3)
Complete agreement between the 6 examinations about the presence of peripheral retinal folds (%)	*No*	13 (21.7)
	*Yes*	47 (78.3)

**Table 3:** Image quality and presence of macular retinal fold according to post-mortem interval

Post-mortem interval	Image quality					Macular retinal fold			
	Insufficient	Discord	Sufficient	Total	Rate of sufficient image quality (%)	No	Yes	Total	Rate of macular retinal fold (%) *P* value = 0,0083
≤ 6h	0	0	12	12	100.0	6	5	11	45.5
6h–12h	2	2	22	26	84.6	5	19	24	79.2
12h–18h	0	0	8	8	100.0	0	8	8	100.0
> 18h	4	2	8	14	57.1	0	9	9	100.0

**Fig. 2:**
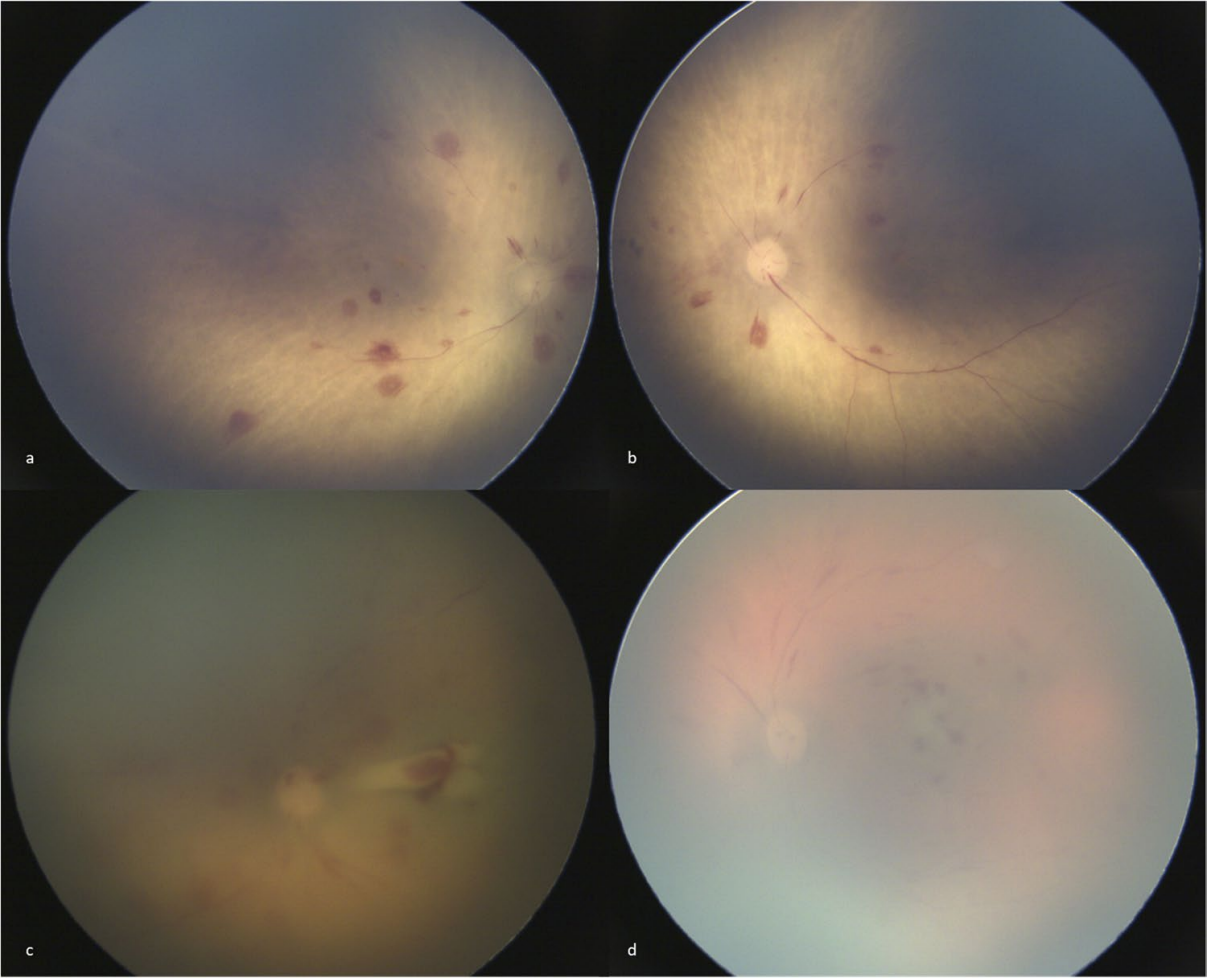
Post-mortem fundus photographs of retinal hemorrhages. **a**, **b** Right and left eyes of a 4-month-old boy, few bilateral superficial retinal hemorrhages confined to the posterior pole (1Bi and 1Ai) and nonspecific of abusive head trauma (AHT), 3 h of post-mortem interval (PMI), undetermined cause of death; (**C**) Left eye of a 1-week-old girl, numerous unilateral retinal hemorrhages (2Bi) nonspecific of AHT and explained by birth, 11 h of PMI, deceased by asphyxia; **d** Left eye of a 1-week-old girl, bilateral retinal hemorrhages (1Ai and 2Bi) nonspecific of AHT and explained by birth, 4 h of PMI, undetermined cause of death

**Fig. 3:**
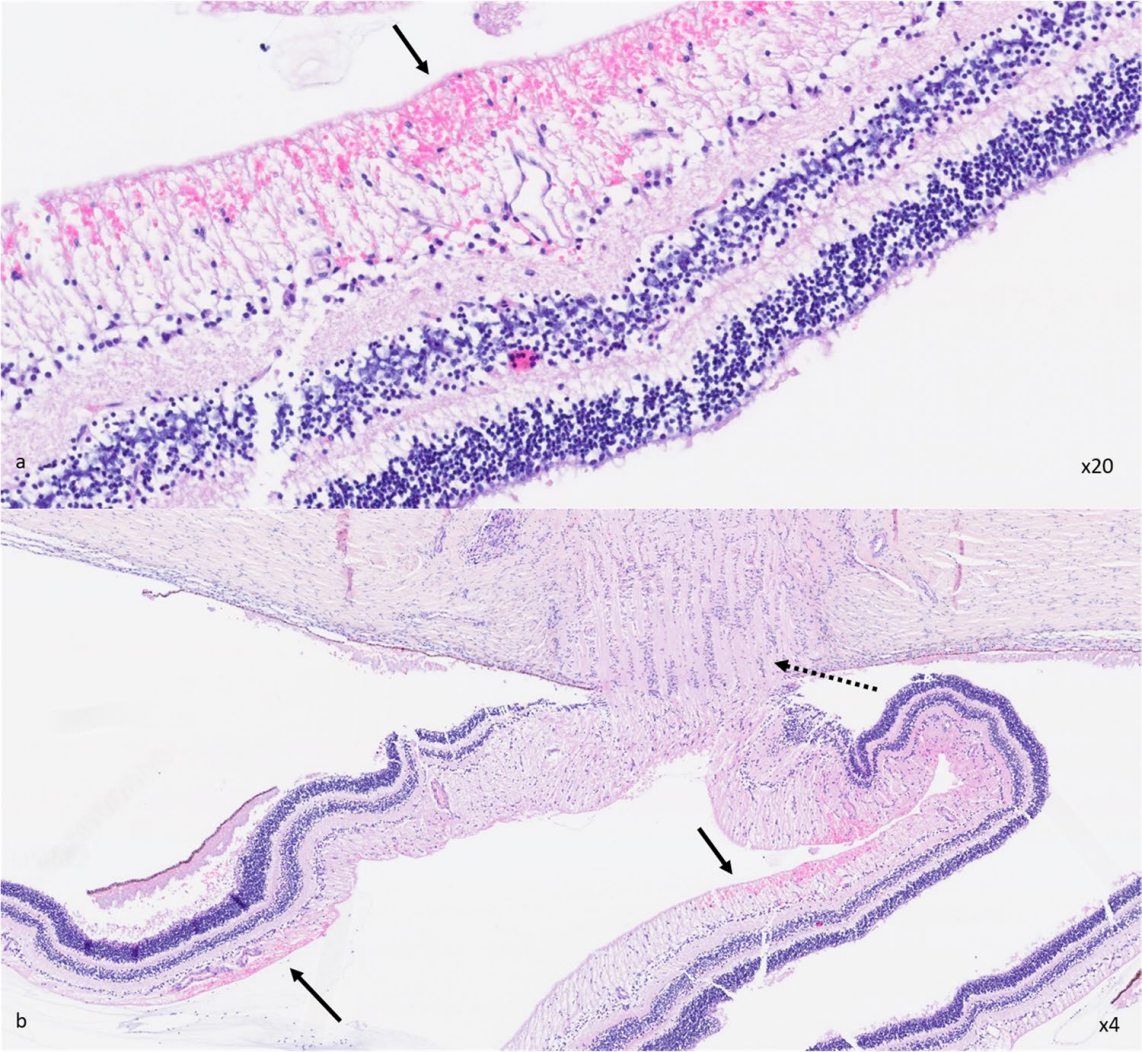
Pathological eye examination with retinal hemorrhages. Pathological examination of the right eye of the 4-month-old boy (undetermined cause of death) with few superficial retinal hemorrhages presented in Fig. 2a and b, nonspecific of abusive head trauma. Microscopic examination of the retina stained with hemalun-eosine (**a**, magnification × 20; **b**, magnification × 4) confirmed the presence of superficial retinal hemorrhages under the inner limiting membrane (black arrows). Dotted arrow shows the optic nerve

RH were found in six eyes (10 %) of four children (13%) (Figs. 2 and 3). The assessment of presence or absence of RH was completely identical between the six examinations for 58 eyes (97%) but was conflicting in two eyes (3%). For these two eyes, the six examinations of the contralateral eye were completely identical. Moreover, the majority was able to categorize the first eye as “not-having RH” and the second as “having RH”. Intra- and inter-raters’ Cohen Kappa led to an excellent to perfect concordance when assessing presence of HR (*κ* = 0.91 [0.74–1.00] to *κ* = 1.00 [1.00–1.00]). Regarding the children with RH, the examination of the death scene and the post-mortem investigations on the children found no evidence for child abuse. In particular, none of the children presented intracranial hemorrhages, bones, or soft tissue traumatic injuries on post-mortem imaging and/or autopsy. Toxicological tests were negative, except for drugs related to resuscitation. None of them presented medical background which could interfere with fundus; in particular, none presented coagulation disorders. They all received prolonged specialized cardiopulmonary resuscitation, with orotracheal intubation, central veinous catheter, and chest compression. Three of the children were admitted between 4 and 96 h in intensive care unit after a transient recovery of cardiac activity. Furthermore, two children were 1 week of age, and therefore, RH could be explained by birth. The two other children were 3 and 4 months old and had few hemorrhages, compatible but not specific for AHT and classified as 1Ai or 1Bi according to the “traumatic hemorrhagic retinopathy (THR) grading system”[17]. The retina was sufficiently visible on the images to affirm that hemorrhages were mostly confined to the posterior pole, only intraretinal, without retinoschisis and few in number (less than 15). Table 4 presents an overview of these different results.

**Table 4:** Clinical information of the children with retinal hemorrhages

Case	Age	Resuscitation	Position of discovery	Extra ocular signs of abusive head trauma*	Cause of death	Laterality of RH	Pathological examination of the eyes	RH Classification at Retcam **
1	6 days	Yes, on the death scene; orotracheal intubation, central venous catheter	Covered by an adult in a sofa	No	Asphyxiation	Unilateral	NO	2Bi
2	6 days	Yes, admission for 24 h in intensive care unit	On the back	No	Undetermined	Bilateral	NO	RE: 1,A,i LE: 2Bi
3	4 months	Yes, admission for few hours in Intensive care unit	Prone position	No	Undetermined	Bilateral	yes superficial intraretinal only hemorrhages confined to the posterior pole (discreet suffusion under the inner limiting membrane), no optic nerve damage	RE: 1,B,i LE: 1,A,i
4	3 months	Yes, admission for 96 h in intensive care unit	Prone position	No	Asphyxiation	Unilateral	no	1,A,i

*At post mortem imaging and autopsy: uni or bilateral subdural hematoma, rupture of bridging veins, skull and/or ribs fractures, other traumatic injury

**Classification criteria follow the “traumatic hemorrhagic retinopathy (THR) grading system” [17]

*1,A,i* less than 10 hemorrhages, intraretinal only, no retinoschisis, one region involved

*1,B,i* more than 10 hemorrhages, intraretinal only, no retinoschisis, one region involved

*2,B,i* more than 10 hemorrhages, intraretinal only, no retinoschisis, two regions involved

*RH* retinal hemorrhages; *RE* right eye; *LE* left eye

**Fig. 4:**
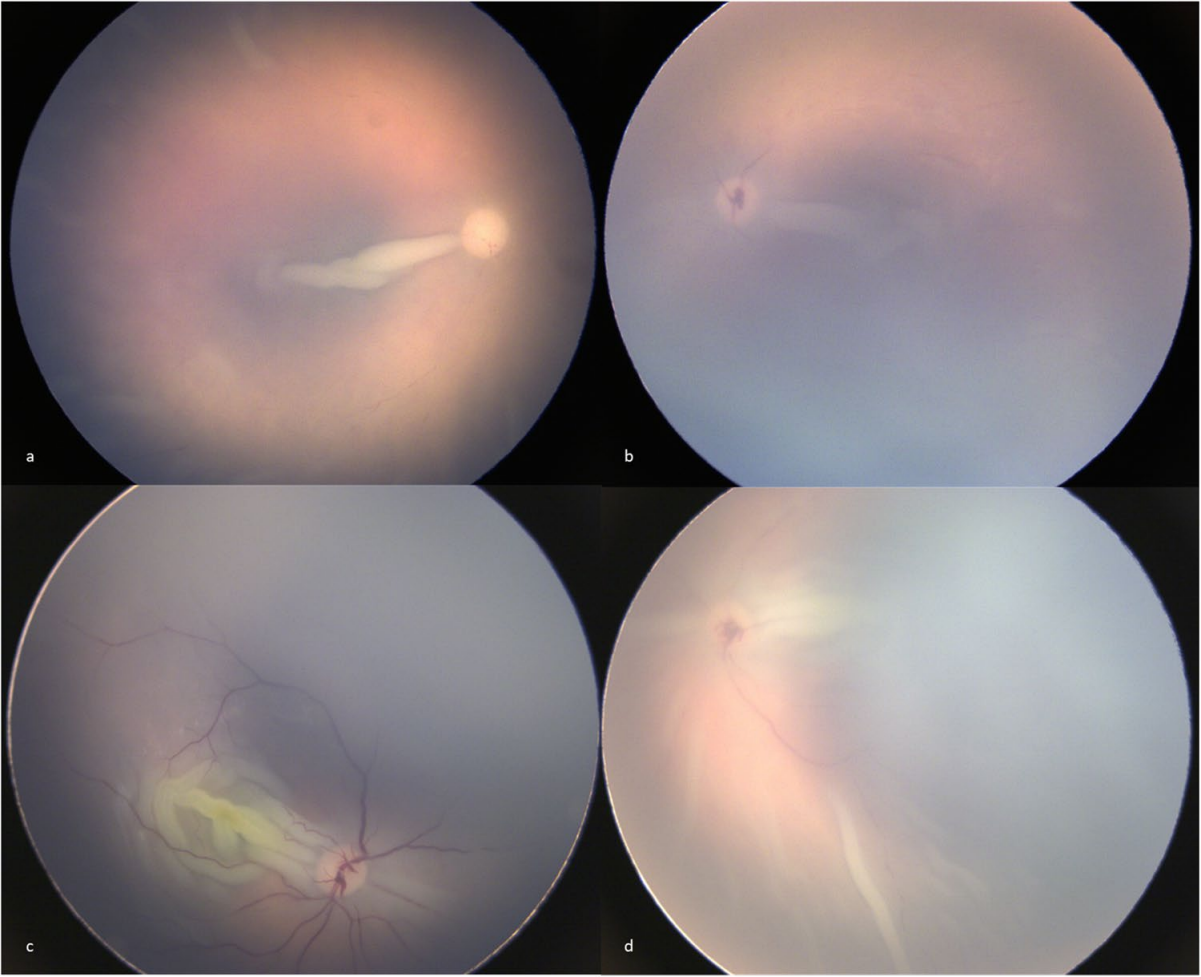
Post-mortem fundus photographs of macular retinal folds, peripheral retinal folds and papillary vessels enlargement. **a** Right eye of a three-month-old boy, unique, long, and thin macular retinal fold, peripheral retinal folds, 27 h of post-mortem interval (PMI), deceased by asphyxia; **b** Left eye of a 3-month-old girl, unique, long and thin macular retinal fold, papillary vessels enlargement, 18 h of PMI, deceased by asphyxia; **c** Left eye of a 18-month-old boy, multiple, long, and large macular retinal folds, 9 h of PMI, sudden infant death syndrome; **d** Left eye of a 15-week-old boy, multiple, long and large macular retinal folds, peripheral retinal folds, papillary vessels enlargement, 11 h of PMI, undetermined cause of death

On PMFP, two post-mortem artifacts were found: macular and peripheral retinal folds and papillary vessels enlargement (Fig. 4, Table 2). Of 52 eyes with image quality sufficient to assess macular fold, median PMI was significantly higher in cases with macular fold (11.3h [8.6–17.8], min = 3.8h, max = 27.3h, *n* = 41) than without (4.5h [4.0–7.5], min = 3.9h, max = 8.8h, *n* = 11, *p* = 0.0003). Macular fold was significantly correlated with PMI and was always present 9 h after death (Table 3).

## Discussion

In this pilot study, image quality of RetCam PMFP in case of SUDI was sufficient to assert presence or absence of RH suggestive of AHT in 83 % of all cases, in 91% when fundus was performed within 18h after death and 67 % (*p*= 0.0096) when performed more than 18h after death. To our knowledge, this is the first study to document the ability of RetCam to detect RH after death. This result can have a major impact on daily practice because PMFP represents a relevant systematic screening test complementary to pathological examination that is still the gold standard.

Eye examination is recommended in case of SUDI to detect RH suggestive of AHT. The American Academy of Pediatrics recommends pathological eyes and orbital tissues examination under 5 years of age if the child has not “clearly died of witnessed severe accidental head trauma or readily diagnosed systemic medical conditions”[8]. But this approach has limitations, the main one being the necessity of eye and orbit tissues removal. If no judicial inquiry is open, parental consent is required and the acceptability of this invasive sampling may be weak for both clinicians and families, even if everything is done to make the change in facial appearance minimal. The technique of eye and orbital tissue removal without disfigurement has been well described [10], but it requires training and regular practice by the forensic pathologist to be performed correctly: this may be an important limitation compounding the fact that SUDI is a rare condition requiring rapid management in order to not aggravate the parental trauma. Moreover, eye enucleation, fixation, and dissection result in artifacts that may alter interpretation: tissue disarrangement, dislocation, retinal detachment, separation of retinal layers, and mechanical tissue damage [10, 19, 20]. Another limitation is the need of a trained ocular pathologist to examine the tissues so that the sample may need to be transported and the response time that may be long. Considering these limitations, pediatricians and forensic pathologists can be tempted to perform eye examination only in some cases, thus depriving all SUDI of systematic screening for RH.

Post-mortem endoscopy has been described by three publications [11, 12, 21]. It provides images of good quality because it circumvents the opacification of cornea and lens. It does not require eye removal, but it is still invasive: it can cause retinal damage leading to artifacts and can alter the presentation of the body at the funeral. Of the 150 cases of post-mortem endoscopy described, it was rarely done in children and performed in only one case of SUDI [11]. No systematic screening in case of SUDI has been described or even proposed. And for good reason: this invasive method has been used by few operators and is limited by the need of an endoscope that is barely used in daily practice in forensic pathology and ophthalmology. Indirect ophthalmoscopy associated with smartphone image acquisition has been previously described but the “do it yourself” association of smartphone and lens is not ergonomic and requires training and practice to be performed correctly [13]. Moreover, no case series has been shown, and data regarding the image quality with indirect ophthalmoscopy is lacking: in our personal experience, post-mortem indirect ophthalmoscopy is difficult, frequently limited by opacification of cornea and lens, whereas visibility is better with a contact camera such as the RetCam. The use of a fundus camera requires a very short learning curve without specific ophthalmologic background: fundus photographs with RetCam can efficiently be performed by nurses or other non-ophthalmologists [22, 23]. Retinal area evaluated with RetCam in pediatric children is significantly higher than with indirect ophthalmoscopy [24].

The quality of the images is a crucial issue; however, it may be difficult to affirm if the quality is sufficient to assert the absence of RH suggestive of SUDI. That notwithstanding, the risk of missing RH suggestive of AHT is low as they present as typically bilateral, extended to the whole fundus, too numerous to count, both intra- and pre retinal and sometimes associated with retinoschisis [8, 16]. In our series, even when image quality seemed low, non-specific and relatively few RH were well visible without any doubt (Fig. 2); incidentally, intra- and inter-raters’ reliability to assert RH, assessed by Cohen’s Kappa, was almost perfect in our series (0.91 to 1.00), with 97% of complete agreement between the six interpretations of PMFP of each eye. To help interpretation of PMFP, it is recommended to compare them with available RetCam photographs of living children suffering from AHT [14] and with our normal RetCam PMFP that are, to our knowledge, available for the first time in the literature. When PMFP show RH or when any doubt persists about the quality of the images, it is recommended to remove the eye to perform pathological examination. In our series, the quality was not sufficient in only 17% of cases, mainly when PMI was superior to 18 h.

The main limits of our study were the lack of AHT cases, and the lack of systematic pathological examination that is still the gold standard to detect post-mortem RH. The number of cases may seem small, but is relatively high considering the low incidence of SUDI. The lack of AHT cases is compensated by the accurate visualization of non-specific RH in three eyes (two children) and RH explained by birth in three other eyes (two children). The correct documentation of these few RH strengthens the hypothesis of the capacity of RetCam to detect RH suggestive of AHT. However, a longitudinal cohort study of PMFP in French cases of SUDI, including more cases and AHT cases with the support of the French registry of SUDI OMIN [25] is necessary. The comparison with pathological examination could be addressed by systematic eye removal for pathological examination when a doubt persists on the quality of PMFP.

To prevent any misuse of this data in court, we wish to stipulate that the purpose of this research was not to discuss the specificity of RH for the diagnosis of AHT. We recommend the reading of well-established literature for the interpretation of RH [8, 16]. In our current practice, the presence of RH was always considered as a sign of possible AHT: this diagnose was finally excluded after all forensic investigations, i.e., medical history, post-mortem imaging, autopsy, and pathological examination.

## Conclusion

In total, this retrospective pilot study has shown that RetCam PMFP offers many advantages that make it a relevant screening test to be carried out as soon as the deceased child arrives at the hospital. PMFP should be done urgently because image quality decreases rapidly after death. This non-invasive examination can be performed by either ophthalmologists, forensic pathologists, pediatricians, or nurses, thanks to its short learning curve and its broad availability in hospitals with a neonatal service. It does not require eye removal and may become a relevant systematic screening test complementary to pathological examination that is still the gold standard. Further studies including more cases, AHT cases, and pathological examinations are needed to define the best decision algorithm to detect RH in case of SUDI.

## Supplementary information

ESM 1 Table 5 Intra- and inter-raters’ concordance to assess the quality of the image and the presence of retinal hemorrhages. Concordance’s level according to Cohen’s Kappa: no agreement (Kappa<0), slight agreement (0 – 0.20), fair agreement (0.21 – 0.40), moderate (0.41 – 0.60), substantial (0.61 – 0.80) or almost perfect (0.81 – 1). (DOCX 15 kb)

## Abbreviations

AHT: Abusive head trauma
IQR: Interquartile range
OCT: Optical coherence tomography
OMIN: Observatoire national des morts inattendues du nourrisson
PMFP: Post-mortem fundus photographs
RH: Retinal hemorrhages
SIDS: Sudden infant death syndrome
SUDI: Sudden unexpected death in infancy

## References

[1] de Visme S, Chalumeau M, Levieux K (2020). National variations in recent trends of sudden unexpected infant death rate in Western Europe. J Pediatr.

[2] Fleming PJ, Blair PS, Pease A (2015). Sudden unexpected death in infancy: aetiology, pathophysiology, epidemiology and prevention in 2015. Arch Dis Child.

[3] Krous HF (2004). Sudden infant death syndrome and unclassified sudden infant deaths: a definitional and diagnostic approach. Pediatrics.

[4] Christian CW, Block R, Committee on Child Abuse and Neglect, American Academy of Pediatrics (2009). Abusive head trauma in infants and children. Pediatrics.

[5] Parks, Annest, Karch (2012) Pediatric abusive head trauma: recommended definitions for public health surveillance and research. https://stacks.cdc.gov/view/cdc/26243

[6] Tursz A, Crost M, Gerbouin-Rérolle P, Cook JM (2010). Underascertainment of child abuse fatalities in France: retrospective analysis of judicial data to assess underreporting of infant homicides in mortality statistics. Child Abuse Negl.

[7] Bhardwaj G, Chowdhury V, Jacobs MB (2010). A systematic review of the diagnostic accuracy of ocular signs in pediatric abusive head trauma. Ophthalmology.

[8] Christian CW, Levin AV, Neglect C on CAA et al (2018) The eye examination in the evaluation of child abuse. Pediatrics 142. 10.1542/peds.2018-1411 10.1542/peds.2018-1411 30037976

[9] Haute Autorité de la Santé (2008). Prise en charge en cas de mort inattendue du nourrisson (moins de 2 ans). J Pédiatrie Puériculture.

[10] Gilliland MGF, Levin AV, Enzenauer RW (2007). Guidelines for postmortem protocol for ocular investigation of sudden unexplained infant death and suspected physical child abuse. Am J Forensic Med Pathol.

[11] Davis N, Wetli C, Shakin J (2006). The retina in forensic medicine: applications of ophthalmic endoscopy: the first 100 cases. Am J Forensic Med Pathol.

[12] Tsujinaka M, Bunai Y (2006). Postmortem ophthalmologic examination by endoscopy. Am J Forensic Med Pathol.

[13] Lantz PE, Schoppe CH, Thibault KL, Porter WT (2016). Smartphone image acquisition during postmortem monocular indirect ophthalmoscopy. J Forensic Sci.

[14] Oliva A, Grassi S, Cazzato F et al (2022) The role of retinal imaging in the management of abusive head trauma cases. Int J Leg Med. 10.1007/s00414-021-02750-5 10.1007/s00414-021-02750-5 35072750

[15] (2007) Haute Autorité de Santé - Prise en charge en cas de mort inattendue du nourrisson (moins de 2 ans). https://www.has-sante.fr/jcms/c_533467/fr/prise-en-charge-en-cas-de-mort-inattendue-du-nourrisson-moins-de-2-ans

[16] Hansen JB, Killough EF, Moffatt ME, Knapp JF (2018). Retinal hemorrhages: abusive head trauma or not?. Pediatr Emerg Care.

[17] Bhardwaj G, Jacobs MB, Martin FJ (2014). Grading system for retinal hemorrhages in abusive head trauma: clinical description and reliability study. J AAPOS Off Publ Am Assoc Pediatr Ophthalmol Strabismus.

[18] R Core Team (2022) R: a language and environment for statistical computing. https://www.R-project.org/

[19] Sorden SD, Larsen T, McPherson LE (2021). Spontaneous background and procedure-related microscopic findings and common artifacts in ocular tissues of laboratory animals in ocular studies. Toxicol Pathol.

[20] Stefani FH, Hasenfratz G (2012). Macroscopic ocular pathology: an Atlas including correlations with standardized echography.

[21] Amberg R, Pollak S (2001). Postmortem endoscopy of the ocular fundus. A valuable tool in forensic postmortem practice. Forensic Sci Int.

[22] Athikarisamy SE, Lam GC, Ross S (2020). Comparison of wide field imaging by nurses with indirect ophthalmoscopy by ophthalmologists for retinopathy of prematurity: a diagnostic accuracy study. BMJ Open.

[23] Murakami Y, Silva RA, Jain A (2010). Stanford University network for diagnosis of retinopathy of prematurity (SUNDROP): 24-month experience with telemedicine screening. Acta Ophthalmol.

[24] Ramkumar H, Koduri M, Conger J (2019). Comparison of digital widefield retinal imaging with indirect ophthalmoscopy in pediatric patients. Ophthalmic Surg Lasers Imaging Retina.

[25] Levieux K, Patural H, Harrewijn I (2018). The French prospective multisite registry on sudden unexpected infant death (OMIN): rationale and study protocol. BMJ Open.

