# Supplementary material for: Assessing retinal hemorrhages with non-invasive post-mortem fundus photographs in sudden unexpected death in infancy
Source: Int J Legal Med. 2023 Feb 23;137(3):913–23. doi: 10.1007/s00414-023-02964-9 (PMC10085933; doi:10.1007/s00414-023-02964-9)
Supplement: Supplementary file 1 — Table 5 Intra- and inter-raters’ concordance to assess the quality of the image and the presence of retinal hemorrhages. Concordance’s level according to Cohen’s Kappa: no agreement (Kappa<0), slight agreement (0 – 0.20), fair agreement (0.21 – 0.40), moderate (0.41 – 0.60), substantial (0.61 – 0.80) or almost perfect (0.81 – 1). (DOCX 15 kb) [file 414_2023_2964_MOESM1_ESM.docx]

| **Is the quality of the image sufficient to assert the presence or absence of retinal hemorrhages?**  **(*Cohen's Kappa 2 by 2*)** | | | | |  |
| --- | --- | --- | --- | --- | --- |
|  |  |  |  |  |  |
|  |  | ***Rater 1*** | ***Rater 2*** | ***Rater 3*** |  |
| **STEP 1** | ***Rater 1*** | **0,71 [0,47 - 0,95]** | - | - |  |
|  | ***Rater 2*** | 0,84 [0,66 - 1,00] | **0,41 [0,12 - 0,70]** | - |  |
|  | ***Rater 3*** | 0,66 [0,39 - 0,93] | 0,53 [0,25 - 0,80] | **0,45 [0,08 - 0,82]** |  |
|  |  | ***Rater 1*** | ***Rater 2*** | ***Rater 3*** |  |
| **STEP 2** | ***Rater 1*** | **0,71 [0,47 - 0,95]** | - | - |  |
|  | ***Rater 2*** | 0,61 [0,33 - 0,89] | **0,41 [0,12 - 0,70]** | - |  |
|  | ***Rater 3*** | 0,58 [0,29 - 0,87] | 0,82 [0,57 - 1,00] | **0,45 [0,08 - 0,82]** |  |
| **If the quality of the image is sufficient, is there any retinal hemorrhages?**  **(Cohen's Kappa 2 by 2)** | | | | |  |
|  |  |  |  |  |  |
|  |  | ***Rater 1*** | ***Rater 2*** | ***Rater 3*** |  |
| **STEP 1** | ***Rater 1*** | **1,00 [1,00 - 1,00]** | - | - |  |
|  | ***Rater 2*** | 1,00 [1,00 - 1,00] | **1,00 [1,00 - 1,00]** | - |  |
|  | ***Rater 3*** | 0,91 [0,74 - 1,00] | 1,00 [1,00 - 1,00] | **0,91 [0,74 - 1,00]** |  |
|  |  | ***Rater 1*** | ***Rater 2*** | ***Rater 3*** |  |
| **STEP 2** | ***Rater 1*** | **1,00 [1,00 - 1,00]** | - | - |  |
|  | ***Rater 2*** | 1,00 [1,00 - 1,00] | **1,00 [1,00 - 1,00]** | - |  |
|  | ***Rater 3*** | 0,81 [0,56 - 1,00] | 0,81 [0,56 - 1,00] | **0,91 [0,74 - 1,00]** |  |
